# Circulating miRNAs Are Associated with the Systemic Extent of Atherosclerosis: Novel Observations for miR-27b and miR-146

**DOI:** 10.3390/diagnostics11020318

**Published:** 2021-02-16

**Authors:** Tiago Pereira-da-Silva, Patrícia Napoleão, Marina C. Costa, André F. Gabriel, Mafalda Selas, Filipa Silva, Francisco J. Enguita, Rui Cruz Ferreira, Miguel Mota Carmo

**Affiliations:** 1 Department of Cardiology, Hospital de Santa Marta, Centro Hospitalar Universitário de Lisboa Central, 1169-024 Lisbon, Portugal; mafalda.selas@gmail.com (M.S.); felipafernandes@gmail.com (F.S.); cruzferreira@netcabo.pt (R.C.F.); 2 NOVA Medical School, Faculdade de Ciências Médicas, Universidade NOVA de Lisboa, 1169-056 Lisbon, Portugal; 3 Instituto de Medicina Molecular João Lobo Antunes, Universidade de Lisboa, 1649-028 Lisbon, Portugal; napoleao.patricia@gmail.com (P.N.); marinacosta@medicina.ulisboa.pt (M.C.C.); andre.gabriel@medicina.ulisboa.pt (A.F.G.); fenguita@medicina.ulisboa.pt (F.J.E.); 4 Cardiomics Unit, Centro Cardiovascular da Universidade de Lisboa, Faculdade de Medicina, Universidade de Lisboa, 1649-028 Lisbon, Portugal; 5 Chronic Diseases Research Center (CEDOC), NOVA Medical School, Faculdade de Ciências Médicas, Universidade NOVA de Lisboa, 1150-082 Lisbon, Portugal; mabmc@sapo.pt

**Keywords:** atherosclerosis, carotid artery disease, coronary artery disease, lower extremity arterial disease, miRNA, miR-27b, miR-146

## Abstract

The mechanisms that regulate the systemic extent of atherosclerosis are not fully understood. We investigated whether the expression of circulating miRNAs is associated with the extent of stable atherosclerosis to a single territory or multiple territories (polyvascular) and with the severity of atherosclerosis in each territory. Ninety-four participants were prospectively recruited and divided into five age- and sex-matched groups: presenting no atherosclerosis, isolated coronary atherosclerosis, coronary and lower extremity atherosclerosis, coronary and carotid atherosclerosis, and atherosclerosis of the coronary, lower extremity, and carotid territories. The expression of six circulating miRNAs with distinct biological roles was assessed. The expression of miR-27b and miR-146 differed across groups (*p* < 0.05), showing a decrease in the presence of atherosclerosis, particularly in the three territories. miR-27b and miR-146 expression decreased in association with a higher severity of coronary, lower extremity, and carotid atherosclerosis. Polyvascular atherosclerosis involving the three territories was independently associated with a decreased miR-27b and miR-146 expression. Both miRNAs presented an area under the curve of ≥0.75 for predicting polyvascular atherosclerosis involving the three territories. To conclude, miR-27b and miR-146 were associated with the presence of severe polyvascular atherosclerosis and with the atherosclerosis severity in each territory. Both are potential biomarkers of severe systemic atherosclerosis.

## 1. Introduction

The mechanisms that regulate the extent of atherosclerosis in a single territory or multiple territories (polyvascular), and the severity of atherosclerosis in each territory, are not fully understood [1,2]. Acquired cardiovascular risk factors and genetic Mendelian inheritance explain only partially the heterogeneity of the systemic extent of stable atherosclerosis [2,3]. Epigenetic mediators, such as miRNAs, which regulate genetic expression at the post-transcriptional level, may contribute to such heterogeneity [4].

The role of several miRNAs in the regulation of different biological pathways involved in atherosclerosis development and progression have been described using preclinical models [4]. Altered expression of different circulating miRNAs in the presence of stable atherosclerosis of specific territories has been identified in humans [5]. However, to the best of our knowledge, there are no published data on the relationship between the expression of circulating miRNAs and the extent of stable atherosclerosis in multiple territories with a systematic screening of atherosclerosis in different territories. Polyvascular atherosclerosis warrants special attention not only because it may have a pathophysiology different from that of single-vascular atherosclerosis, but also because it is frequently encountered in clinical practice and is associated with a higher risk of ischemic events [6,7]. Knowledge on the miRNA expression profile in polyvascular atherosclerosis may provide insights into its phenotype, and therefore, its pathophysiology, since the biological role of several miRNAs has been identified in vitro [4]. Moreover, investigating a potential signature of circulating miRNAs in polyvascular atherosclerosis may be useful for clinical practice as it could contribute to identifying simple non-invasive diagnostic biomarkers for the stratification of systemic atherosclerotic burden [4].

The principal aim of this study was to determine whether the expression of circulating miRNAs is associated with the extent of stable atherosclerosis in a single-vascular or polyvascular disease. We also investigated whether the expression of circulating miRNAs is associated with atherosclerosis severity in each territory. We hypothesized that the miRNA expression profile is associated with the systemic extent of atherosclerosis in multiple territories and the severity of atherosclerosis in each territory.

## 2. Materials and Methods

This study is a part of a project aimed at assessing the mechanisms underlying the expression of stable atherosclerosis as a single-vascular (coronary) or polyvascular (coronary, lower extremity, and/or carotid) disease. The study protocol was approved by the ethics committees of the involved institutions (Centro Hospitalar Universitário de Lisboa Central, Nr. 245/2015, in 2015, and NOVA Medical School, Faculdade de Ciências Médicas, Universidade NOVA de Lisboa, Nr. 000176, in 2015). The investigation conformed to the principles outlined in the Helsinki Declaration. All the participants signed informed consent forms.

### 2.1. Recruitment of Participants

To assess the expression levels of circulating miRNAs in single-vascular and polyvascular atherosclerosis, we prospectively recruited five groups of age- and sex-matched participants from our center: control, with no coronary, lower extremity, or carotid atherosclerosis; group 1, with isolated coronary atherosclerosis; group 2, with coronary and lower extremity atherosclerosis; group 3, with coronary and carotid atherosclerosis; and group 4, with atherosclerosis of the coronary, lower extremity, and carotid territories. All the participants were screened for obstructive atherosclerotic disease in the three territories.

Coronary artery disease was excluded in control participants if they presented no effort angina; no evidence of coronary atherosclerosis on coronary computed tomography angiography (64-slice computed tomography scanner LightSpeed VCT XT, GE Healthcare, Milwaukee, WI, USA), including a calcium score of 0 and no soft plaques; and no positive myocardial stress test (the latter was not mandated to be assessed as per protocol). In other participants, coronary artery disease was defined as luminal stenosis of at least 50% for the left main artery or at least 70% for other epicardial vessels on invasive coronary angiography. Lower extremity arterial disease was defined as a significant (≥50%) stenosis on Doppler ultrasound at rest [8,9] or the combination of chronic claudication and an ankle–brachial index equal to or less than 0.9 [9,10]. Doppler ultrasound was performed for the characterization of lower extremity arterial disease. Carotid artery disease was defined as a stenosis of at least 50% on Doppler ultrasound [9,10]. All Doppler ultrasound studies of the lower extremity and carotid arteries were performed according to a standardized protocol, using the Logiq S7 Expert Ultrasound System (GE Healthcare, Wauwatosa, WI, USA), and measurements were performed while following published guidelines [9,10,11].

The exclusion criteria were as follows: patients with acute ischemic events within 12 months, either coronary, lower extremity, or cerebrovascular events; those with coronary artery bypass grafting or lower extremity bypass surgery performed within 12 months; those with prior carotid endarterectomy or prior percutaneous intervention of the coronary, lower extremity, or carotid arteries; those with critical limb ischemia (with ischemic rest pain), heart failure, hemodynamically significant valvular heart disease, hematological disorders, active infection, history of malignancy, chronic kidney disease (stage 4 or 5), or severe hepatic dysfunction; those under 18 years of age; and those unable or unwilling to consent to study participation.

### 2.2. Sample Size

No previous studies reported data on circulating miRNAs in both single-vascular and polyvascular atherosclerosis with the systematic screening of atherosclerotic lesions in different territories, which would be valuable for supporting the sample size estimation in our study. In this pilot study, we planned to recruit at least 20 control participants, 20 patients with isolated coronary atherosclerosis (group 1), and 40 patients with coronary and extra-coronary atherosclerosis (groups 2 to 4), including at least 10 patients per group in groups 2 to 4. The recruitment of participants for each group continued even after meeting the minimum number of participants until the minimum sample size was achieved for all groups.

### 2.3. Data Collection

Data were collected prospectively after patient inclusion. A standardized record of clinical, demographic, laboratory, echocardiographic, Doppler ultrasound, computed tomography angiography, and invasive coronary angiography data was obtained from each participant.

To evaluate the severity of coronary atherosclerosis, the following parameters were assessed, based on invasive coronary angiography: the number of vessels with obstructive disease (the left main artery, left anterior descending artery, circumflex artery, and right coronary artery were scored separately, with a total score ranging from 0 to 4), the number of coronary artery lesions, and the synergy between percutaneous coronary intervention with taxus and cardiac surgery (SYNTAX) score [12], using acknowledged methods [12,13]. To evaluate the severity of lower extremity atherosclerosis, the number of arterial segments with obstructive disease on both sides was assessed, including the external iliac, common femoral, superficial femoral, popliteal, anterior tibial, posterior tibial, and fibular arteries [9,14]. The number of affected carotid sides was assessed.

### 2.4. Candidate miRNAs

Six candidate miRNAs (miR-21, miR-27b, miR-29a, miR-126, miR-146, and miR-218), each playing a distinct biological role associated with the regulation of atherosclerosis development and expression, were selected [4,13,14]. Among other functions, miR-21 regulates vascular smooth cell and endothelial cell functions; miR-27b regulates lipid metabolism, development of lipid-induced atherosclerotic lesions, and inflammatory pathways; miR-29a regulates fibrosis and extracellular matrix composition; miR-126 regulates endothelial function in response to shear stress; miR-146 regulates endothelial function in response to inflammatory cytokines; and miR-218 regulates endothelial cell migration [4,15,16].

### 2.5. Quantification of Expression Levels of Candidate miRNAs

Peripheral blood was collected early in the morning under fasting conditions. Serum was separated by centrifugation (500× *g* for 10 min) within 15 min of sampling. Aliquots were stored at −80 °C and samples were thawed only once.

Total RNA was extracted from serum samples using the miRCURY™ RNA Isolation Kit (Qiagen, Hilden, Germany). Complementary DNA was synthesized from total RNA using the Universal cDNA synthesis kit from miRCURY™ LNA miRNA system (Qiagen, Hilden, Germany). MiRNA amplification was performed using quantitative reverse-transcription polymerase chain reaction (using the miRCURY™ LNA SYBR Green PCR Kit and LNA™ PCR primers, Qiagen, Hilden, Germany), and the melting curve was determined according to the following conditions: 95 °C for 10 min followed by 45 cycles of 95 °C for 10 s and 60 °C for 60 s. All the reactions were performed in triplicates. The amplification data were assessed using DataAssist™ Software v3.01 (Thermo Fisher Scientific, Waltham, MA, USA). Cycle threshold (Ct) values greater than 40 were considered undetermined [17,18,19,20]. The relative expression levels of the six candidate miRNAs were calculated using the delta Ct (ΔCt) method, normalizing for the UniSp6 RNA spike-in control [20,21,22,23]. Higher ΔCt miRs represent lower circulating levels of the candidate miRNAs [20,21,22,23].

### 2.6. Statistical Analysis

Discrete variables are presented as frequencies (percentages) and continuous variables are presented as the mean (standard deviation) in normally distributed data or median (interquartile range (IQR)) in variables without a normal distribution (Shapiro–Wilk test). Categorical variables were analyzed using the chi-square or Fisher’s exact tests. Continuous variables were analyzed using Student’s *t*-test or the Mann–Whitney test when the normality was not verified. Comparisons between multiple groups were performed using the analysis of variance (ANOVA) for normally distributed data and Kruskal–Wallis test for variables without a normal distribution; the Bonferroni post hoc correction was used for multiple pairwise comparisons. Pearson’s correlation was used to test correlations between continuous variables. Multivariate linear regression analysis was performed using all clinical, laboratory, and atherosclerosis data (including the systemic extent of atherosclerosis, severity of atherosclerosis in different territories, and prior revascularization) as independent variables, and ΔCt miR-27b and ΔCt miR-146 as dependent variables. Variables with a *p*-value of <0.10 in the univariate analysis were tested in the multivariate model, with a correction for collinearity. The areas under the receiver operating characteristic (ROC) curves of ΔCt miR-27b and ΔCt miR-146 for predicting severe polyvascular atherosclerosis (involving the three territories) were assessed. The level of significance was set at α = 0.05. Analyses were conducted using SPSS software, version 26.0 (IBM Corp, Armonk, NY, USA).

## 3. Results

### 3.1. Clinical Characteristics, Laboratory Results, and Atherosclerosis Data of Participants

Ninety-four participants were included: 26 control participants, 20 with isolated coronary atherosclerosis (group 1), 18 with coronary and lower extremity atherosclerosis (group 2), 12 with coronary and carotid atherosclerosis (group 3), and 18 with atherosclerosis of the coronary, lower extremity, and carotid territories (group 4). The clinical characteristics, laboratory results, and atherosclerosis data of the participants are presented in Table 1. The differences in clinical characteristics and laboratory data across groups were driven by controls, which showed a lower prevalence of classical cardiovascular risk factors and use of antiplatelet and statin therapy, and lower neutrophil count and creatinine levels. Globally, the distribution of clinical and laboratory parameters did not differ among groups 1 to 4. The severity of coronary atherosclerosis did not differ among groups 1 to 4, including the number of diseased vessels, number of coronary lesions, and the SYNTAX score (median 3, IQR 2–4; median 4, IQR 3–5; and mean 26.5 [9.3], respectively, among all patients with coronary atherosclerosis). The number of lower extremity lesions was higher in group 4 than in group 2 (median 3, IQR 2–5, among all patients with lower extremity atherosclerosis). Among the patients with carotid atherosclerosis, 37% had bilateral disease, and this rate did not differ between groups 3 and 4.

### 3.2. Expression of Circulating miRNAs According to the Systemic Extent of Atherosclerosis

The expression levels (ΔCt) of miR-27b and miR-146 differed across groups (ANOVA *p* < 0.05 for both miRNAs; Table 2), whereas the expression levels of other miRNAs did not.

**Figure 1:**
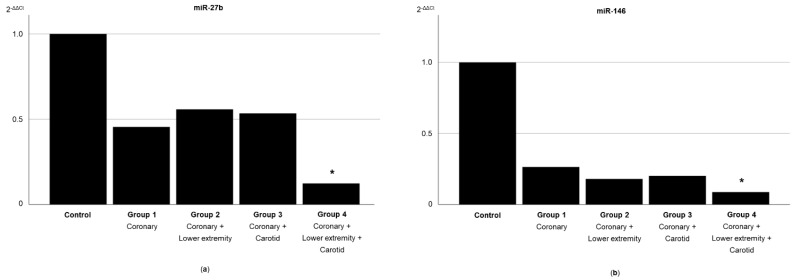
Relative expression of miR-27b and miR-146 according to the systemic extent of atherosclerosis in different territories. The mean relative expression levels of (**a**) miR-27b and (**b**) miR-146 are presented using the Livak method (2^−ΔΔCt^), with lower values corresponding to lower expression levels [24]. * *p*-value < 0.05 vs. controls.

The ΔCt values of both miRNAs showed a stepwise increase (corresponding to a decrease in their expression levels) from control participants to patients with atherosclerosis of one or two territories (groups 1–3), and further to patients with atherosclerosis of three territories (group 4), with significant differences between the controls and patients with atherosclerosis of three territories. The relative expression of miR-27b and miR-146 is presented in Figure 1 [24].

### 3.3. Expression of Circulating miRNAs According to the Atherosclerosis Severity in Different Territories

For both miR-27b and miR-146, there was a weak positive correlation between ΔCt miR and the number of coronary arteries with obstructive disease (*r* = 0.241, *p* = 0.043 for miR-27b; and *r* = 0.242, *p* = 0.036 for miR-146), number of coronary artery lesions (*r* = 0.241, *p* = 0.043 for miR-27b; and *r* = 0.289, *p* = 0.012 for miR-146), and the SYNTAX score (*r* = 0.286, *p* = 0.019 for miR-27b; and *r* = 0.257, *p* = 0.037 for miR-146), indicating decreased expression levels of both miRNAs with an increase in the severity of coronary atherosclerosis.

For miR-27b, miR-126, and miR-146, there was a weak positive correlation between ΔCt miR and the number of obstructive lesions in the lower extremity (*r* = 0.320, *p* = 0.008 for miR-27b; *r* = 0.254, *p* = 0.043 for miR-126; and *r* = 0.352, *p* = 0.003 for miR-146), indicating decreased expression levels of the three miRNAs with an increase in the severity of lower extremity atherosclerosis.

ΔCt miR-27b showed a stepwise increase from individuals with no carotid atherosclerosis (18.6 [3.9]) to patients with unilateral disease (19.6 [5.9]), and further to patients with bilateral disease (21.7 [4.9]), with a significant difference between individuals with no carotid atherosclerosis and those with bilateral disease (*p* = 0.030), corresponding to lower expression levels of miR-27b in bilateral carotid atherosclerosis.

### 3.4. Atherosclerosis Severity and miR-27b and miR-146 Expression Levels in Multivariate Analysis

The univariate analysis assessing the parameters associated with ΔCt miR-27b and ΔCt miR-146 is presented in Supplementary Tables S1 and S2, respectively (Supplementary Materials). In the multivariate linear regression analysis, the presence of polyvascular atherosclerosis involving the coronary, lower extremity, and carotid territories, and indexes of coronary artery disease severity were independently associated with ΔCt miR-27b and ΔCt miR-146 (Table 3), after adjusting for the clinical characteristics, laboratory results, and other parameters of atherosclerosis severity.

### 3.5. miR-27b and miR-146 as Predictors of Polyvascular Atherosclerosis

**Figure 2:**
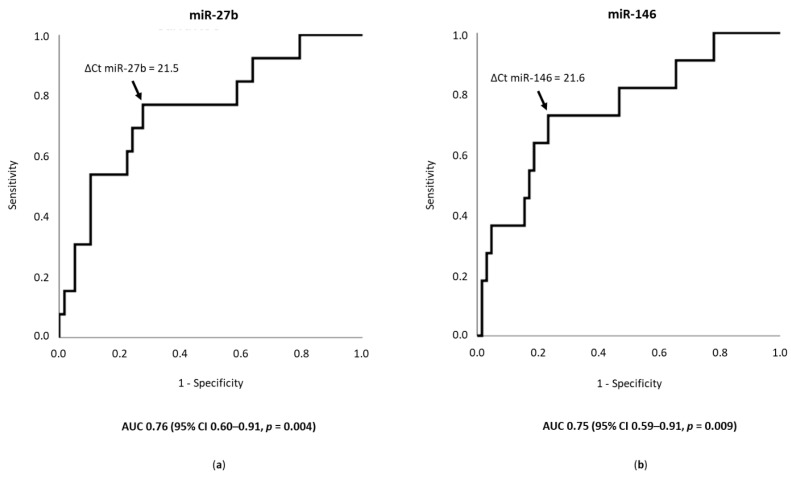
Accuracy of miR-27b and miR-146 as predictors of polyvascular atherosclerosis. The receiver operating characteristics curve is presented for (**a**) ΔCt miR-27b and (**b**) ΔCt miR-146, as predictors of polyvascular atherosclerosis involving the coronary, lower extremity, and carotid territories. The optimal cut-off for (**a**) ΔCt miR-27b was 21.5 and for (**b**) ΔCt miR-146 was 21.6, presenting a Youden’s index [25,26] of 0.49 and 0.50, respectively. 95% CI—95% confidence interval; AUC—area under the curve.

ΔCt miR-27b and ΔCt miR-146 provided areas under the ROC curve of 0.76 (95% confidence interval [CI] 0.60–0.91, *p* = 0.004) and 0.75 (95% CI 0.59–0.91, *p* = 0.009), respectively, for predicting polyvascular atherosclerosis with concomitant involvement of the coronary, lower extremity, and carotid arteries (Figure 2). The cut-off (21.5) of ΔCt miR-27b indicated 77% sensitivity and 72% specificity, and the cut-off (21.6) of ΔCt miR-146 indicated 73% sensitivity and 77% specificity for predicting polyvascular atherosclerosis with concomitant involvement of the three territories.

## 4. Discussion

In this prospective study, four main findings stood out: lower expression levels of miR-27b and miR-146 were associated with an increase in the systemic extent of atherosclerosis to multiple territories; lower expression levels of miR-27b and miR-146 were associated with an increase in the atherosclerosis severity within different arterial territories; polyvascular atherosclerosis involving the coronary, lower extremity, and carotid territories was independently associated with the miR-27b and miR-146 expression levels; and both miR-27b and miR-146 were reasonably accurate in predicting severe polyvascular atherosclerosis involving the three territories. The observed association between circulating miRNAs and the severity of atherosclerosis, particularly its systemic extent, is discussed below, along with an overview of published data on miRNA profiles in polyvascular atherosclerosis and potential clinical implications of the results. The roles of miR-27b and miR-146 in the regulation of atherosclerosis in experimental studies and the associations of both miRNAs with atherosclerosis expression in humans are also discussed.

To the best of our knowledge, this is the first study assessing the circulating miRNA expression profile in single- and polyvascular atherosclerosis with a systematic screening of atherosclerotic lesions in three major territories of atherosclerosis. One study reported an altered miRNA expression profile based on the number of territories with manifestations of atherosclerosis [21]. However, in this study, patients had heart failure, which may have altered the expression levels of several miRNAs [21,27], and the definition of atherosclerotic manifestation included prior acute ischemic events or revascularization procedures, without a systematic screening of atherosclerotic lesions in the corresponding territory [21]. We observed that polyvascular atherosclerosis was associated with a specific miRNA expression profile, particularly in severe polyvascular atherosclerosis with simultaneous involvement of the coronary, lower extremity, and carotid territories. In such a scenario, both the atheroprotective miR-27b and miR-146 were significantly downregulated. Consistently, the expression levels of these two miRNAs decreased with an increase in the severity of atherosclerosis in different territories, as assessed by different complementary indexes [9,12,28]. The systemic extent of atherosclerosis with involvement of the three territories was a major determinant of miR-27b and miR-146 expression levels in multivariate analysis. Moreover, miR-27b and miR-146 showed reasonable accuracy for predicting severe systemic atherosclerosis with involvement of the three territories. The results highlight miR-27b and miR-146 as potential biomarkers that can contribute to detect polyvascular atherosclerosis non-invasively. Screening of polyvascular atherosclerosis in daily clinical practice by assessing simultaneously different arterial territories using currently available methods is potentially laborious [9,28]. Therefore, miR-27b and miR-146 may simplify the stratification of systemic atherosclerotic burden [9,28]. Considering the high morbidity and risk of mortality associated with polyvascular atherosclerosis [6,7], the identification of severe systemic atherosclerosis using a simple, non-invasive tool may be clinically useful as it could facilitate an early intensification of the atheroprotective regimens, such as antithrombotic and lipid-lowering therapies [29,30,31,32,33]. Further studies are needed to confirm these hypotheses.

In experimental studies, complementary atheroprotective mechanisms of miR-27b have been reported, including the prevention of atherosclerosis by suppression of lipoprotein lipase–induced lipid accumulation and inflammatory response in mice [34]; downregulation of the expression of key genes involved in lipid metabolism and mitigation of the accumulation of lipids in circulation [35]; decrease in vascular inflammation by the suppression of pro-inflammatory factor release [36], including that of interleukin 17–induced monocyte chemoattractant protein-1 [37], contributing to a decrease in monocyte–macrophage activation [38]; and repression of repulsive semaphorins, thereby facilitating the formation of tight endothelial monolayers and stable vessels in response to shear stress [39]. miR-146 is induced in endothelial cells in response to pro-inflammatory cytokines and acts as a negative feedback regulator of inflammatory signaling in endothelial cells by dampening the activation of pro-inflammatory transcriptional programs, including the NF-κB, AP-1, and MAPK/EGR pathways, and by promoting eNOS expression [4,15,40]. In addition, the enhancement of miR-146a levels in monocytes and macrophages by cellular apoE suppresses NF-κB-mediated inflammation and atherosclerosis [41]. Considering the miR-27b and miR-146 dysregulation observed in our study and their specific biological roles described in the aforementioned experimental studies [4,15,16,34,35,36,37,38,39,40,41], the extent of atherosclerosis in multiple territories and within each territory appears to be influenced by lipid metabolism and lipid accumulation in the vessel, and by the inflammatory response involving endothelial and monocyte–macrophage cells. These data reinforce the described mechanistic role of miRNAs in coupling lipid metabolism, endothelial dysfunction, inflammation, and atherosclerosis [42,43,44,45,46]. The interplay is complex, with each miRNA acting in multiple pathways and in different steps of a specific pathway, as post-transcriptional hubs.

In humans, published data on miR-27b and miR-146 expression in the presence of atherosclerosis are scarce. miR-27b was reported to be downregulated in the presence of lower extremity atherosclerosis [47]. Although miR-146 has been reported to be upregulated in patients with coronary atherosclerosis [48], which conflicts with the experimental data [4,13,30], it was reported to be downregulated in more severe forms of coronary atherosclerosis, including acute coronary syndromes, compared with stable coronary artery disease [49], and coronary atherosclerosis associated with poor collateral circulation [50]. No data have been published on the expression levels of these two miRNAs in polyvascular disease. As previously discussed, not only data from experimental studies but also studies in humans support the downregulation of miR-27b and miR-146 in more severe forms of atherosclerosis, including polyvascular atherosclerosis.

The strengths of this study should be acknowledged. To the best of our knowledge, this is the first description of the expression profile of circulating miRNAs based on the presence of single-vascular and polyvascular obstructive atherosclerosis. The prospective nature of this study and the systematic screening of three major arterial territories have contributed to the accuracy of our findings. The consistency of results was reinforced for the following reasons: the multivariate analysis carried out confirmed the independent association between polyvascular atherosclerosis and miR-27b and miR-146 expression; analyses of the severity/extent of atherosclerosis within different territories showed a similar direction of miR-27b and miR-146 dysregulation compared with the analyses of the systemic extent of atherosclerosis (specifically, miR-27b and miR-146 downregulation in more severe presentations of atherosclerosis); and the results are in line with data from experimental studies and studies in patients with atherosclerosis [4,15,16,34,35,36,37,38,39,40,41,47,49,50]. This is the first report on the diagnostic accuracy of circulating miRNAs, including miR-27b and miR-146, for the detection of severe polyvascular atherosclerosis. The results have potential implications for a clinical condition that is frequent in clinical practice and associated with considerable morbimortality, as both miRNAs may consist of simple non-invasive tools for stratifying the systemic atherosclerotic burden.

Our study has some limitations. The sample may be of limited size. However, as this study pioneered the investigation of the expression of circulating miRNAs in single-vascular and polyvascular atherosclerosis with a systematic screening of atherosclerotic lesions in different territories, no data were available to support the sample size estimation. In this exploratory pilot study, the sample size was sufficient to analyze the miRNA expression profile in such a clinical scenario, allowing to detect differences in the expression levels of circulating miRNAs in polyvascular atherosclerosis. The atherosclerosis extent in multiple territories and disease severity within each territory are commonly associated, and both may influence miRNA expression levels [4,21,51], which could have been a potential source of bias regarding the studied association between atherosclerosis extent to multiple territories and miRNA expression. However, coronary artery disease, which was the common factor to all patients, presented a well-balanced severity among the different groups of patients. Moreover, the multivariate analysis confirmed an independent association between the atherosclerosis extent in multiple territories and miRNA (miR-27b and miR-146) expression levels, irrespective of disease severity within each territory. This suggests that atherosclerosis severity within each territory may not have been a significant source of bias. Of note, this is a single-center study, which may limit the applicability of results to different clinical settings. Further multicentric studies performing an external validation are warranted.

## 5. Conclusions

Lower expression levels of atheroprotective miR-27b and miR-146 were associated with the presence of severe polyvascular atherosclerosis and with higher severity of atherosclerosis in different territories, including the coronary, lower extremity, and carotid territories. The simultaneous involvement of the three territories was the most important determinant of miR-27b and miR-146 dysregulation, and both miRNAs showed reasonable accuracy for predicting polyvascular atherosclerosis affecting the three territories. Our results provide insights into the pathophysiology of polyvascular atherosclerosis. In addition, miR-27b and miR-146 seem to be promising non-invasive biomarkers for refining the stratification of systemic atherosclerotic burden and, therefore, may contribute to the tailoring of primary prevention strategies.

## Figures and Tables

**Table 1:** Characteristics of the participants according to the involved territories of atherosclerosis.

Study Group	Control	Group 1	Group 2	Group 3	Group 4	*p*-Value
Territories of atherosclerosis	None	Coronary	Coronary + LE	Coronary + Carotid	Coronary + LE + Carotid	
*n*	26	20	18	12	18	
**Clinical Characteristics**						
Age, years	59 (53–69)	65 (56–70)	67 (57–72)	59 (51–73)	69 (60–75)	0.079
Male, *n* (%)	23 (88.5)	18 (90.0)	16 (88.9)	10 (83.3)	17 (94.4)	0.912
Hypertension, *n* (%)	14 (53.8)	17 (85.0) ^a^	18 (100.0) ^a^	11 (91.7) ^a^	18 (100.0) ^a^	<0.001
SBP, mmHg	132 (14)	130 (20)	133 (21)	135 (21)	130 (18)	0.939
DBP, mmHg	73 (10)	72 (11)	72 (11)	73 (12)	65 (9)	0.175
Dyslipidemia, *n* (%)	18 (69.2)	19 (95.0) ^a^	18 (100.0) ^a^	11 (91.7)	17 (94.4) ^a^	0.010
Diabetes mellitus, *n* (%)	3 (11.5)	6 (30.0)	8 (44.4) ^a^	6 (50.0) ^a^	9 (50.0) ^a^	0.036
Smoking history, *n* (%)	6 (23.1)	9 (45.0)	12 (66.7) ^a^	4 (33.3)	12 (66.7) ^a^	0.014
LVEF > 50%, *n* (%)	26 (100.0)	20 (100.0)	18 (100.0)	12 (100.0)	18 (100.0)	–
Antiplatelet therapy, *n* (%)	6 (23.1)	20 (100.0) ^a^	17 (94.4) ^a^	11 (91.7) ^a^	18 (100) ^a^	<0.001
Statin therapy, *n* (%)	13 (50.0)	18 (90.0) ^a^	16 (94.1) ^a^	11 (91.7) ^a^	16 (88.9) ^a^	0.001
**Laboratory Parameters**						
Hemoglobin, g/dL	13.9 (12.9–15.0)	14.53 (10.0–15.1)	14.1 (13.2–14.6)	12.0 (11.4–13.4) ^b^	12.9 (12.1–14.2)	0.017
Leukocyte count, 10^9^/L	6.4 (1.7)	7.4 (1.9)	7.3 (1.7)	7.5 (2.2)	8.1 (1.7)	0.080
Neutrophil count, 10^9^/L	3.2 (2.5–4.8)	4.1 (3.4–5.2)	3.9 (3.4–4.8)	4.0 (3.4–6.7)	4.7 (3.6–6.0) ^a^	0.043
Lymphocyte count, 10^9^/L	1.9 (1.7–2.2)	2.1 (1.6–2.4)	2.1 (1.6–2.8)	1.7 (1.2–2.3)	2.2 (1.6–2.6)	0.401
Platelet count, 10^9^/L	242 (191–274)	209 (176–269)	219 (195–264)	229 (137–251)	227 (203–263)	0.854
Fasting glycaemia, mg/dL	89 (80–98)	94 (86–129)	94 (83–125)	99 (84–157)	85 (75–123)	0.385
Percentage of glycosylated hemoglobin	5.6 (5.2–5.9)	5.9 (5.6–6.7)	5.9 (5.5–6.1)	5.8 (5.4–7.4)	5.9 (5.3–7.7)	0.185
Creatinine, mg/dL	0.8 (0.7–0.9)	0.9 (0.8–1.1)	0.8 (0.8–1.2)	0.9 (0.8–1.4)	1.1 (0.9–1.5) ^a^	0.002
Total cholesterol, mg/dL	186 (51)	164 (38)	172 (50)	153 (50)	173 (49)	0.329
LDL-cholesterol, mg/dL	99 (77–141)	95.0 (71–120)	106 (83–120)	65 (56–132)	117 (82–142)	0.297
HDL-cholesterol, mg/dL	51 (44–58)	35 (31–41) ^a^	35 (31–45) ^a^	40 (27–44) ^a^	40 (32–42) ^a^	<0.001
Triglycerides, mg/dL	106 (67–144)	142 (98–206)	115 (83–204)	100 (62–177)	117 (95–171)	0.423
C-reactive protein, mg/L	4.1 (2.0)	3.7 (1.4)	3.8 (1.1)	4.1 (2.3)	3.3 (2.0)	0.151
**Coronary Artery Disease**						
Nr. of vessels with obstructive disease *	0 (0–0)	3 (2–3) ^a^	3 (2–4) ^a^	3 (3–3) ^a^	3 (2–4) ^a^	<0.001
Nr. of lesions	0 (0–0)	4 (2–5) ^a^	4 (3–5) ^a^	4 (3–5) ^a^	4 (3–5) ^a^	<0.001
SYNTAX score	0 (0–0)	23.3 (8.4) ^a^	29.6 (9.9) ^a^	25.3 (7.2) ^a^	28.1 (10.6) ^a^	<0.001
Prior CABG, *n* (%)	0 (0.0)	7 (35.0) ^a^	7 (38.9) ^a^	6 (50.0) ^a^	3 (16.7) ^a^	0.002
**LE Arterial Disease**						
Bilateral disease, *n* (%)	0 (0.0)	0 (0.0)	10 (55.5) ^a,b,d,e^	0 (0.0)	15 (83.3) ^a–d^	<0.001
Nr. of segments with obstructive disease	0 (0–0)	0 (0–0)	2 (1–4) ^a,b,d,e^	0 (0–0)	4 (3–5) ^a–d^	<0.001
Prior bypass surgery, *n* (%)	0 (0.0)	0 (0.0)	2 (11.1) ^a,b,d^	0 (0.0)	6 (33.3) ^a,b,d^	<0.001
**Carotid Artery Disease**						
Bilateral disease, *n* (%)	0 (0.0)	0 (0.0)	0 (0.0)	3 (25.0) ^a–c^	8 (44.4) ^a–c^	<0.001

Categorical variables are expressed as frequency (percentage) and continuous variables as the mean (standard deviation) or median (interquartile range). CABG—coronary artery bypass grafting; DBP—diastolic blood pressure; HDL—high-density lipoproteins; LDL—low-density lipoproteins; LE—lower extremity; LVEF—left ventricular ejection fraction; Nr.—number; SBP—systolic blood pressure; SYNTAX—synergy between percutaneous coronary intervention with taxus and cardiac surgery. ^a^ *p*-value < 0.05 vs. controls; ^b^ *p*-value < 0.05 vs. group 1; ^c^ *p*-value < 0.05 vs. group 2; ^d^ *p*-value < 0.05 vs. group 3; ^e^ *p*-value < 0.05 vs. group 4; * the left main artery, left anterior descending artery, circumflex artery, and right coronary artery were scored separately, with a total score ranging from 0 to 4.

**Table 2:** Expression of circulating miRNAs according to the involved territories of atherosclerosis.

Study Group	Control	Group 1	Group 2	Group 3	Group 4	*p*-Value
Territories of atherosclerosis	None	Coronary	Coronary + LE	Coronary + Carotid	Coronary + LE + Carotid	
**miRNAs**						
miR-21	14.73 (4.63)	15.60 (4.22)	14.14 (4.41)	13.83 (5.06)	18.22 (4.01)	0.064
miR-27b	17.82 (3.43)	19.39 (4.06)	19.03 (4.37)	18.71 (5.45)	22.34 (3.93) ^a^	0.041
miR-29a	20.90 (3.52)	21.09 (2.02)	20.67 (2.90)	20.99 (5.06)	23.50 (2.79)	0.121
miR-126	17.38 (15.12–21.89)	17.79 (14.99–22.88)	16.79 (15.57–22.74)	14.85 (13.36–21.34)	23.32 (17.88–24.86)	0.102
miR-146	18.06 (3.00)	19.63 (3.34)	19.43 (4.39)	18.76 (4.21)	22.20 (3.47) ^a^	0.048
miR-218	22.63 (10.01–23.51)	23.15 (16.18–26.12)	22.63 (10.87–25.71)	22.67 (9.19–23.36)	24.70 (23.88–25.68)	0.744

Delta Ct (ΔCt) values are presented for each miRNA (higher ΔCt values correspond to lower miRNA expression levels) and are expressed as the mean (standard deviation) or median (interquartile range). LE—lower extremity. ^a^ *p*-value < 0.05 vs. controls.

**Table 3:** Parameters associated with miR-27b and miR-146 expression levels in multivariate linear regression analysis.

	β	95% CI	*p*-Value
**ΔCt miR-27b**			
Coexistence of coronary, LE, and carotid atherosclerosis	3.41	0.55–6.27	0.020
SYNTAX score	0.072	0.001–0.143	0.049
**ΔCt miR-146**			
Coexistence of coronary, LE, and carotid atherosclerosis	3.10	0.73–5.46	0.011
Number of coronary artery lesions	0.373	0.015–0.730	0.041

95% CI—95% confidence interval; LE—lower extremity; SYNTAX—synergy between percutaneous coronary intervention with taxus and cardiac surgery.

## Data Availability

The data presented in this study are available on request from the corresponding author. The data are not publicly available due to personal data protection.

## Supplementary Materials

The following are available online at https://www.mdpi.com/2075-4418/11/2/318/s1: Table S1: Association of clinical characteristics, laboratory results, and atherosclerosis data with miR-27b expression levels; Table S2: Association of clinical characteristics, laboratory results, and atherosclerosis data with miR-146 expression levels.
