# Supplementary material for: Circulating miRNAs Are Associated with the Systemic Extent of Atherosclerosis: Novel Observations for miR-27b and miR-146
Source: Diagnostics (Basel). 2021 Feb 16;11(2):318. doi: 10.3390/diagnostics11020318 (PMC7920287; doi:10.3390/diagnostics11020318)
Supplement: Supplementary file 1 [file diagnostics-11-00318-s001.pdf]

Supplementary Materials

Circulating miRNAs Are Associated with the Systemic Extent of Atherosclerosis: Novel Observations for miR-27b and miR-146

Tiago Pereira-da-Silva, Patrícia Napoleão, Marina C. Costa, André F. Gabriel, Mafalda Selas, Filipa Silva, Francisco J. Enguita, Rui Cruz Ferreira and Miguel Mota Carmo

Table S1. Association of clinical characteristics, laboratory results, and atherosclerosis data with miR-27b expression levels.

Clinical Data		ΔCt miR-27b	*p*-Value
Age, years [a]		$r = 0.137$	0.254
Sex [b]	Male	19.4 (15.16–23.11)	0.585
	Female	17.88 (13.63–23.24)	
Hypertension [b]	No	19.77 (15.81–22.16)	1.000
	Yes	18.71 (14.96–23.53)	
Systolic blood pressure [a]		$r = -0.076$	0.530
Diastolic blood pressure [a]		$r = -0.174$	0.149
Dyslipidemia [b]	No	19.93 (15.73–21.26)	0.649
	Yes	18.35 (14.97–23.79)	
Diabetes mellitus [b]	No	18.29 (15.75–22.12)	0.377
	Yes	22.09 (14.41–24.10)	
Smoking history [b]	No	19.29 (4.42)	0.892
	Yes	19.15 (4.34)	
Antiplatelet therapy [b]	No	17.03 (14.05–19.85)	0.014
	Yes	20.71 (16.18–23.89)	
Statin therapy [b]	No	17.89 (14.53–20.10)	0.118
	Yes	19.50 (15.33–23.83)	
**Laboratory Data**			
Hemoglobin, g/dL [a]		$r = -0.200$	0.097
Leukocyte count, 10^9/L [a]		$r = 0.197$	0.111
Neutrophil count, 10^9/L [a]		$r = 0.188$	0.122
Lymphocyte count, 10^9/L [a]		$r = 0.081$	0.512
Neutrophil/lymphocyte ratio [a]		$r = 0.132$	0.286
Platelet count, 10^9/L [a]		$r = 0.185$	0.132
Fasting glycaemia, mg/dL [a]		$r = 0.023$	0.849
Percentage of glycosylated hemoglobin [a]		$r = 0.128$	0.311
Creatinine, mg/dL [a]		$r = 0.283$	0.017
Total cholesterol, mg/dL [a]		$r = -0.068$	0.578
LDL-cholesterol, mg/dL [a]		$r = -0.033$	0.788
HDL-cholesterol, mg/dL [a]		$r = -0.123$	0.312
Triglycerides, mg/dL [a]		$r = -0.041$	0.737
C-reactive protein, mg/L [a]		$r = 0.018$	0.889
**Atherosclerosis Data**			
**Coronary Artery Disease**			
Nr. of vessels with obstructive disease [a]		$r = 0.241$	0.043
Nr. of obstructive lesions [a]		$r = 0.241$	0.043

SYNTAX score [a]		r = 0.286	0.019
Prior coronary artery bypass grafting [b]	No	20.68 (18.16–21.22)	0.301
	Yes	18.89 (16.27–19.04)	
**LE Arterial Disease**			
Nr. of sides affected [b]	One	20.35 (15.17–23.26)	0.041
	Two	24.02 (17.23–25.74)	
Nr. of segments with obstructive disease [a]		r = 0.320	0.008
Prior bypass surgery [b]	No	21.90 (15.51–23.92)	0.498
	Yes	22.06 (16.50–24.34)	
**Carotid Artery Disease**			
Nr. of sides affected [b]	One	19.89 (13.31–24.51)	0.857
	Two	23.41 (17.17–24.49)	

[a] Correlations between miR-27b expression levels (ΔCt miR-27b) and continuous variables were tested and the correlation coefficient (r) is presented for each; [b] miR-27b expression levels (ΔCt miR-27b) were compared between groups for categorical variables and are expressed as the mean (standard deviation) or median (interquartile range). Higher ΔCt miR-27b represent lower circulating levels of miR-27b. HDL—high-density lipoproteins; LDL—low-density lipoproteins; LE—lower extremity; Nr.—number; SYNTAX—SYNergy between percutaneous coronary intervention with TAXus and cardiac surgery; ΔCt—delta cycle threshold.

Table S2. Association of clinical characteristics, laboratory results, and atherosclerosis data with miR-146 expression levels.

**Clinical Data**		**ΔCt miR-146**	***p*-Value**
Age, years [a]		r = 0.139	0.234
Sex [b]	Male	19.46 (3.75)	0.643
	Female	18.79 (4.54)	
Hypertension [b]	No	19.60 (2.87)	0.833
	Yes	19.35 (3.99)	
Systolic blood pressure [a]		r = −0.081	0.494
Diastolic blood pressure [a]		r = −0.161	0.170
Dyslipidemia [b]	No	19.69 (2.71)	0.814
	Yes	19.35 (3.94)	
Diabetes mellitus [b]	No	19.09 (3.59)	0.357
	Yes	19.95 (4.21)	
Smoking history [b]	No	19.65 (3.61)	0.528
	Yes	19.09 (4.06)	
Antiplatelet therapy [b]	No	17.64 (2.91)	0.031
	Yes	19.90 (3.92)	
Statin therapy [b]	No	18.92 (3.67)	0.434
	Yes	19.72 (3.58)	
**Laboratory Data**			
Hemoglobin, g/dL [a]		r = −0.140	0.236
Leukocyte count, 10^9/L [a]		r = 0.161	0.178
Neutrophil count, 10^9/L [a]		r = 0.169	0.154
Lymphocyte count, 10^9/L [a]		r = 0.031	0.794
Neutrophil/lymphocyte ratio [a]		r = 0.157	0.192
Platelet count, 10^9/L [a]		r = 0.201	0.091
Fasting glycaemia, mg/dL [a]		r = 0.071	0.550
Percentage of glycosylated hemoglobin [a]		r = 0.179	0.142
Creatinine, mg/dL [a]		r = 0.202	0.082
Total cholesterol, mg/dL [a]		r = −0.079	0.505

LDL-cholesterol, mg/dL [a]		$r = -0.043$	0.713
HDL-cholesterol, mg/dL [a]		$r = -0.039$	0.741
Triglycerides, mg/dL [a]		$r = -0.080$	0.501
C-reactive protein, mg/L [a]		$r = -0.100$	0.419
**Atherosclerosis Data**			
**Coronary Artery Disease**			
Nr. of vessels with obstructive disease [a]		$r = 0.242$	0.036
Nr. of obstructive lesions [a]		$r = 0.289$	0.012
SYNTAX score [a]		$r = 0.257$	0.037
Prior coronary artery bypass grafting [b]	No	20.97 (16.94–22.80)	0.209
	Yes	18.34 (15.74–20.81)	
**LE Arterial Disease**			
Nr. of sides affected [b]	One	18.21 (4.47)	0.021
	Two	21.87 (3.51)	
Nr. of segments with obstructive disease [a]		$r = 0.352$	0.003
Prior bypass surgery [b]	No	20.13 (4.17)	0.437
	Yes	21.59 (4.55)	
**Carotid Artery Disease**			
Nr. of sides affected [b]	One	19.58 (4.95)	0.352
	Two	21.40 (3.18)	

[a] Correlations between miR-146 expression levels (ΔCt miR-146) and continuous variables were tested and the correlation coefficient (*r*) is presented for each; [b] miR-146 expression levels (ΔCt miR-146) were compared between groups for categorical variables and are expressed as the mean (standard deviation) or median (interquartile range). Higher ΔCt miR-146 represent lower circulating levels of miR-146. HDL—high-density lipoproteins; LDL—low-density lipoproteins; LE—lower extremity; Nr.—number; SYNTAX—SYNergy between percutaneous coronary intervention with TAXus and cardiac surgery; ΔCt—delta cycle threshold.
